# Analysis of binary mixture of oxytetracycline and bromohexine in their combined veterinary formulation by four simple spectrophotometric methods with greenness assessment

**DOI:** 10.1186/s13065-024-01296-y

**Published:** 2024-10-03

**Authors:** Marco M.Z. Sharkawi, Mark T. Safwat, Nada S. Abdelwahab

**Affiliations:** 1 Pharmaceutical Analytical Chemistry Department, Faculty of Pharmacy, Beni-Suef University, Alshaheed Shehata Ahmed Hegazy St, Beni-Suef, 62514 Egypt; 2 Pharmaceutical Chemistry Department, Faculty of Pharmacy, Nahda University in Beni-Suef (NUB), Beni-Suef, Egypt

**Keywords:** Absorption correction, Bromhexine, Dual wavelength, Induced dual wavelength, Oxytetracycline, Spectrum Subtraction

## Abstract

Tetracyclines family is considered as the first-line antibiotic drugs for food animals. Formulating bromhexine (BR) with oxytetracycline (OTC) improved the antibacterial activity of OTC, besides it is considered as a mucolytic agent. Four precise, rapid, and simple spectrophotometric methods were successfully developed for resolution of the overlapped spectra of OTC and BR in their pure form and in their pharmaceutical formulation. The proposed methods are absorption correction (AC), dual wavelength (DW), induced dual wavelength (IDW), and spectrum subtraction (SS) spectrophotometric methods. The developed methods were used for the determination of OTC and BR in the ranges of 2–50 µg/mL and 1–30 µg/mL, respectively for all methods. For (AC) and (SS) methods, OTC and BR were determined at 380 nm and 245.6 nm, respectively after spectral resolution steps. While for (DW) method, the absorbance difference between λ (271.8 –287.6 nm) and λ (245.6 –283.2 nm) were used for the determination of OTC and BR, in order. For IDW, it depended on using the absorbance difference between 271.8 nm and 245.6 nm as well as the equality factor (F) calculated for each drug at the selected wavelengths. In all methods, HCl was used as a solvent and they are validated according to ICH guidelines. Several green metric tools have been developed to evaluate the greenness of the analytical methods like National Environmental Methods Index (NEMI), Modified NEMI, Analytical Eco-scale, Green Analytical Procedure Index (GAPI) and Analytical GREEnness calculator (AGREE), and all ensured the low impact of the suggested approaches on health and environment. The proposed methods are highly selective, robust and precise. Additionally they are time and money effective and can be used in any analytical laboratory.

**Supplementary Information:**

The online version contains supplementary material available at 10.1186/s13065-024-01296-y.

## Introduction

**Fig. 1:**
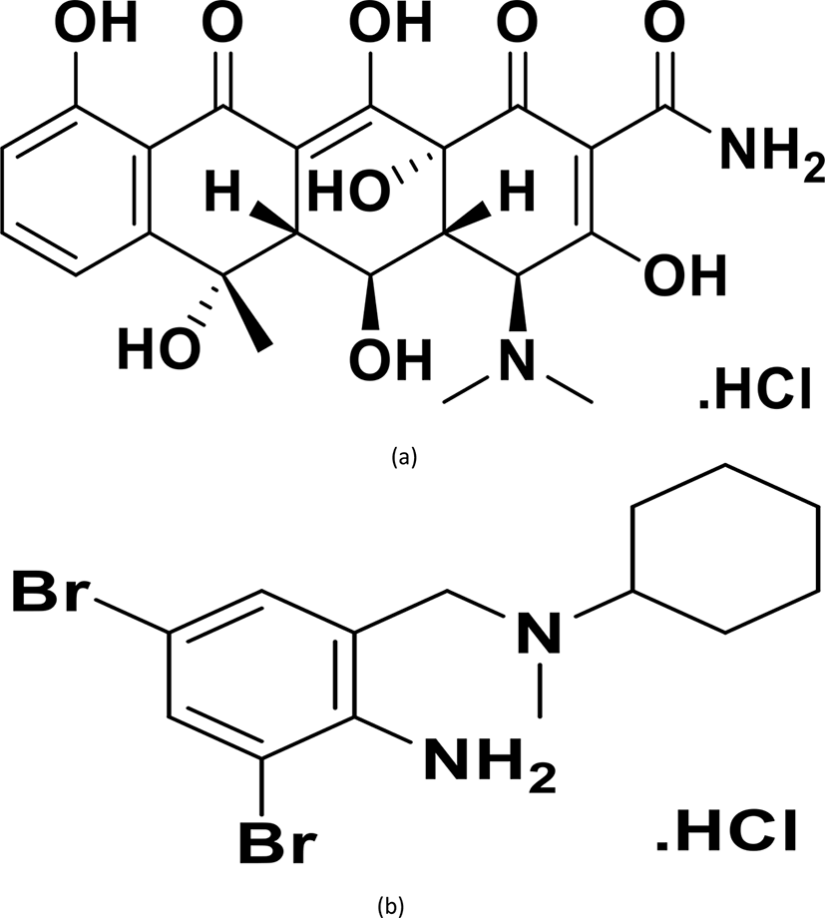
Chemical structure of oxytetracycline hydrochloride (**a**) and bromhexien hydrochloride (**b**)

Tetracyclines have proven to be effective against many pathogenic bacteria. They are used as broad-spectrum antibiotics and as prophylaxis in food animals. They are not expensive and so they are widely used in veterinary world with thousands tons of production yearly [1]. OTC represents an essential member of tetracycline family which is extensively used in veterinary medicines Fig. 1a. It is mainly used for gastrointestinal and respiratory infectious diseases primarily for positive/negative bacteria, rickettsia, mycoplasma, and chlamydia species [2].

Bromhexine hydrochloride (BR) Fig. 1b is a mucolytic agent that decreases sputum thickness to be expelled by coughing. It increases the quantity of OTC eliminated from plasma and produced in sputum, resulting in a considerable rise in OTC activity [3].

According to a literature review, several analytical methods for the analysis of OTC alone or in mixture with other drugs have been reported. Among these methods are spectrophotometric [4–7], TLC [5, 8–10], HPLC [11–13], LC-MS-MS [14, 15], and different electrochemical methods including conductmetric, potentiometric and cyclic voltammetric methods [16]. While BR was determined by spectrophotometric methods [17–21], TLC [22, 23], HPLC [24–27], LC-MS-MS [28, 29] and electrochemical methods [30]. Both OTC and BR were analyzed in their veterinary preparation by HPLC method [31]. On the other hand, in our previously published work, we measured the drugs under investigation in milk samples by TLC and HPLC methods [32]. As observed from the literature review, the binary mixture has been resolved only by using chromatographic methods which need sophisticated apparatus and that are not available in all laboratories as expensive solvents.

In pharmaceutical analysis, spectrophotometry has been widely used and extensively employed due to its simplicity, low expense, easy to use and save time. The disadvantages of spectrophotometry is its low selectivity, the issue which recently can be resolved by using novel spectrophotometric methods that depend on data processing using simple algorithms. These methods such as DW, IDW, AC, and SS methods able to resolve complex spectra with minimal data processing steps and without using complex algorithms or software like chemometry.

Nowadays, Green chemistry is extensively used to make chemistry healthier and ecofriendly. It works on using greener solvents, chemicals and technologies characterized by less or zero harmful environmental impact [33]. Different green tools are concurrently used to estimate the greenness of the methods, each concern with evaluating certain items and parameters on the process. It is difficult to find a metric tool that evaluates the whole process, so it is advisable to use more than one metric to form a true and complete picture about the greenness of the methods. Interestingly, in the present work, a green comparative study was established between the developed spectrophotometric methods and the reported HPLC one [31] using five metrics; National Environmental Methods Index (NEMI) [34], Modified NEMI [35], Analytical Eco-scale [36], Green Analytical Procedure Index (GAPI) [37] and Analytical GREEnness calculator (AGREE) [38] to reach a conclusion discuss the environmental impact of the developed and the reported methods.

The goal of this research is to develop a variety of fast, accurate, selective, reproducible and low-cost spectrophotometric methods, including absorption correction (AC), dual wavelength method (DW), induced dual wavelength method (IDW), and spectrum subtraction method (SS) methods that are used to determine OTC and BR in their binary mixture as well as in their combined dosage form. The developed spectrophotometric methods have the advantages over the reported HPLC [31] method of being greener methods as they guarantee the protection of both researchers and the environment by substituting organic harmful solvents (Trifluroacetic acid and acetonitrile) with more ecofriendly alternatives (aqueous HCl). They also represents a simple way for analyzing the studied components in daily quality control laboratories that do not need well trained persons, sophisticated steps and instruments.

## Experimental

### Samples

#### Oxytetracycline hydrochloride (OTC)

Oxytetracycline hydrochloride (OTC) was kindly supplied by (Pharco pharmaceuticals, Amriya, Alexandria, Egypt), the supplied material has the purity of 99.53%, 100.34%, 99.42% and 99.53% according to the proposed methods; Absorption correction, dual wavelength, induced dual wavelength and spectrum subtraction methods, in order.

#### Bromhexine hydrochloride (BR)

Bromhexine hydrochloride (BR) was purchased from sigma Aldrich (Merck Life Science BV, Ildefonse Vandammestraat 5/7B, 1560 Hoeilaart, BELGIUM) of 98.95% 100.34%, 98.88% and 98.95% purity according to accuracy of the proposed methods: Absorption correction, dual wavelength, induced dual wavelength and spectrum subtraction methods, respectively.

#### Oxyclear

Oxyclear ^®^ injectable solution: (batch number 190317, Pharma Swede, Egypt), labeled to contain OTC 50 mg/mL (Equivalent to 46.3 mg of Oxytetracycline) and BR 3 mg/mL. (Equivalent to 2.74 mg Bromhexine), It was purchased from local Egyptian market.

### Chemicals and solvents

- Chemicals and solvents that were used throughout this work were methanol (Fisher, Southborough, UK) and hydrochloric acid, (Adwic, Cairo, Egypt)
- Distilled water (Sedico Pharmaceutical Co, New Cairo, Cairo, Egypt).

### Instrument

A double beam UV- Visible spectrophotometer (Shimadzu 1800 PC Series Spectrophotometer, Japan), model UV-1601 PC with 1 cm path length quartz cell, connected to compatible computer. UV-PC software version (3.7) was used for all absorbance measurements and data analysis.

### Solutions

#### Preparation of 0.1 mol/L HCl

It was prepared by diluting 10 mL concentrated HCl by distilled water till adjusting the volume to 1000 mL.

#### Stock Standard solutions (1000 µg/mL)

Stock standard solutions each of OTC and BR were prepared by weighing 25 mg of each drug and transferring into two separate 25 mL volumetric flasks, mixing well with about 5 mL of methanol and the volume was then completed to the mark using 0.1 mol/L HCl.

#### Working Standard solutions (100 µg/mL)

Working standard solutions were prepared for each of OTC and BR by separately transferring 5 mL from their respective stock solutions (1000 µg/mL) into two separate 50 mL volumetric flasks and then completing the volume by 0.1 mol/L HCl .

#### Laboratory prepared mixture solutions

Six different laboratory prepared mixtures containing different ratios of OTC and BR of concentration ratios of (25/1.5, 20/20, 40/2, 50/3, 50/5, 30/10 µg/mL, in order), were prepared by adding different volumes from their respective working standard solutions into a set of six 10 mL volumetric flasks and then the volume was adjusted with 0.1 mol/L HCl. Details of the prepared mixtures are presented in Table S.1.

#### Pharmaceutical formulation solutions

Pharmaceutical dosage form stock solution of (2500/150 µg/mL) was prepared by .delivering 1.25 mL of Oxyclear^®^ injectable solution accurately into 25 mL glass volumetric flask then mixing well with 5 mL methanol and the volume was completed aftr that with 0.1 mol/L HCl. Diluted sample of (25/1.5 µg/mL) of OTC and BR, respectively) was obtained from the previously prepared dosage form stock solution by transferring 0.1 mL into 10 mL glass volumetric flask and the final dilution was carried out using 0.1 mol/L HCl.

## Procedure

### Construction of calibration curves

Several dilutions were obtained from the previously prepared working solutions (100 µg/mL) to obtain samples containing OTC and BR separately in the ranges of 2–50 µg/mL and 1–30 µg/mL, respectively using 0.1 mol/L HCl for final dilutions. The spectra of each drug were recorded over the wavelength range of 200–400. Further processing of the stored spectra was done following the instructions of each method for construction of the calibration curves.

#### For OTC determination

##### For absorption correction method

Calibration curve was constructed relating OTC absorbance λ_max_ = 380 nm (at which BR showed no absorbance) to its corresponding concentrations. Moreover, absorptivity factor for OTC was calculated relating its absorbance at 245.6 nm to that at 380 nm (λ_245.6_ / λ_380_).

##### For dual wavelength method

Calibration curve was conducted relating the difference in absorption between λ _(271.8 –287.6 nm)_ (zero difference for BR) to different OTC concentrations.

##### For induced dual wavelength method

The absorbance of OTC at 245.6 nm was recorded and then multiplied by the equality factor of BR (F_BR_ = absorbance of BR at λ _271.8 nm_/λ _245.6 nm_). After that, the absorbance difference between 271.8 nm and 245.6 nm after multiplication by F_BR_ was calculated and the calibration curve was constructed relating the absorbance difference to the corresponding concentrations of OTC.

##### For spectrum subtraction method

Calibration curve was constructed relating absorbance at λmax = 380 nm) to OTC corresponding concentrations.

#### For BR determination

##### For absorption correction and spectrum subtraction methods

Calibration curve was plotted relating absorbance at 245.6 nm to the corresponding BR concentrations.

##### For dual wavelength method

Calibration curve was constructed relating the difference in absorbance difference between 245.6 nm and 283.2 nm (zero difference for OTC) to different BR concentrations.

##### For induced dual wavelength method

The absorbance of BR at 271.8 was recorded and then multiplied by the equality factor of OTC (F_OTC_ = absorbance of OTC at λ _245.6 nm_/λ _271.8 nm_). After that, calibration plot relating the absorbance difference between 245.6 nm and 271.8 nm after multiplying the later by F_OTC_ to BR corresponding concentration was obtained.

After that, regression equations were computed for the studied components.

### Application to the laboratory prepared mixtures

The spectra of the previously prepared mixtures were recorded over the range of 200–400 nm and further data processing was performed to calculate the concentrations of the studied drugs in the prepared mixtures.

#### For absorption correction method

The absorbance of the stored spectra of the mixtures was determined at λ_max_ = 380 nm (corresponding to OTC where BR showed no absorbance) and the concentrations were then calculated using the regression equation for OTC at 380 nm.

While for BR determination in the mixture, the absorbance of the mixture at 380 nm was multiplied by absorptivity factor of OTC (λ_245.6 nm_/ λ_380 nm_). The resulted value corresponding to absorbance contribution of OTC at 245.6 nm, then this value was subtracted from the total absorption of the mixture at 245.6 nm to obtain the absorption of BR at 245.6 nm which was then was used to calculate its concentrations by substitution in the corresponding regression equation.

#### For dual wavelength method

The absorbance difference between 271.8 nm and 287.6 nm in the stored spectra of the mixtures (zero difference for BR) was used for the determination of OTC. After that, the concentration of OTC was computed using the corresponding regression equation.

For BR determination in mixture, the absorbance difference between 245.6 nm and 283.2 nm in (zero difference for OTC) was calculated. After that, the concentrations of BR were then calculated using the corresponding regression equation.

#### For induced dual wavelength method

For OTC determination, the absorbance of each mixture at 245.6 was multiplied by the equality factor of BR (F_BR_ = absorbance of BR at λ _271.8 nm_/λ _245.6 nm_). After that, the absorbance difference between 271.8 and 245.6 nm after multiplication of the later by F_BR_ was calculated and then the resolved value was used to calculate OTC concentration using its corresponding equation.

For the BR determination, the absorbance of mixture at 271.8 nm was multiplied by the equality factor of OTC (F_OTC_ = absorbance of OTC at λ _245.6 nm_/λ _271.8 nm_). After that, the absorbance difference between 245.6 nm and 271.8 nm after multiplication by F_OTC_ was calculated and then the concentration of BR was obtained using its regression equation.

#### For spectrum subtraction method

For OTC determination in mixture, the mixture spectrum was divided by the normalized spectrum of OTC resulting in plateau region. The amplitude value at the plateau region at 380 nm was then multiplied by OTC normalized spectrum resulting in D^0^ spectrum of OTC in mixture. After that, the absorbance at 380 nm was measured and used to calculate OTC concentration using its equation. While for BR, it was determined in mixture by subtracting the previously resolved OTC D^o^ spectrum from the total stored spectrum of the mixture resulting in D^o^ spectrum corresponding to BR. After that, absorbance value at 245.6 nm of the extracted BR spectrum was measured and used for its determination by application in the corresponding regression equation.

### Application to the pharmaceutical formulations

The spectra of the prepared pharmaceutical dosage form sample was recorded from 200 to 400 nm and then the developed methods applied for resolving the laboratory prepared mixtures were followed and the regression equations previously computed were used to calculate the concentrations of the cited drugs. Standard addition technique was performed on three different concentrations of each drug to evaluate method accuracy.

## Result and discussion

It was quite challenging to separate the overlapped spectra of multi-component mixtures without prior separation of the active constituents. Spectrophotometric methods are favored over other expensive and complex instrumental methods like chromatographic methods which are often necessitate the optimization of various conditions such as pH, temperature, and flow rate.

In this work, four simple methods are adopted to resolve the overlapped spectra of OTC and BR. The proposed methods are absorption correction, dual wavelength, induced dual wavelength and spectrum subtraction methods which have the capability to distinguish between these spectra in few, easy and precise steps so they can be used for quality control and routine analysis of the studied mixture.

Different solvents were tried such as methanol, water, 0.1 mol/L HCl and 0.1 mol/L NaOH during methods optimization. 0.1 mol/L HCl was found to be the best solvent for final dilution regarding spectral resolution and method selectivity. Additionally all prepared solutions and samples were found to be stable for at least 1 week in 0.1 M HCL where no spectral changes were observed upon scanning over the range of 200–400 nm.

### Absorption correction method (AC)

This method is useful to solve the overlapped spectra of two drugs in condition that, one of them shows no interference from the other one at certain wavelength. It cannot be used for resolving of complete overlapped spectra [39, 40].

#### Principle

- This method uses the absorbance at two selected wavelengths, one at λ_max_ of the first drug (X) where other drug (Y) also shows considerable absorbance or interference (λ_1_) and the second wavelength (λ_2_) at which the first drug (X) shows no absorbance or interference (λ_2_).

$${{\rm{A}}_{{\rm{\lambda 1}}}} = {\rm{A}}{{\rm{X}}_{{\rm{\lambda 1}}}} + {\rm{A}}{{\rm{Y}}_{{\rm{\lambda 1}}}} \ldots \ldots {{\rm{A}}_{{\rm{\lambda 2}}}} = {\rm{A}}{{\rm{Y}}_{{\rm{\lambda 2}}}}$$

Where A_λ1_ is the absorbance at λ_1_ where the drug X shows interference with Y and it used to determine drug X. while A_λ2_ is the absorbance at λ_2_ where drug X shows no interference with Y and it used to determine drug Y.

#### For drug (X) determination

1. Absorption factor for drug (Y) is calculated which is the ratio between absorbance at (λ_1_) and (λ_2_) ( F_Y_ = λ_1_/ λ_2_).
2. Multiplying the absorption of the mixture at λ_2_ by the previously calculated absorption factor (F_Y_), giving the absorbance relating to drug (Y) at λ_1_ where it interferes with component (x).
3. Subtraction of the obtained value in step 2 from the total mixture absorbance at λ_1_ giving the absorbance corresponding to component (x) in the binary mixture.

#### For drug (Y) determination in mixture

- The absorbance of drug Y at λ_2_ is used to determine its concentration in the binary mixture (drug X show s no interference). Then concentrations of X and Y in the mixture are determined using regression equations relating the absorbance of pure standards of either X or Y at the selected wavelengths to their corresponding concentrations.

**Fig. 2:**
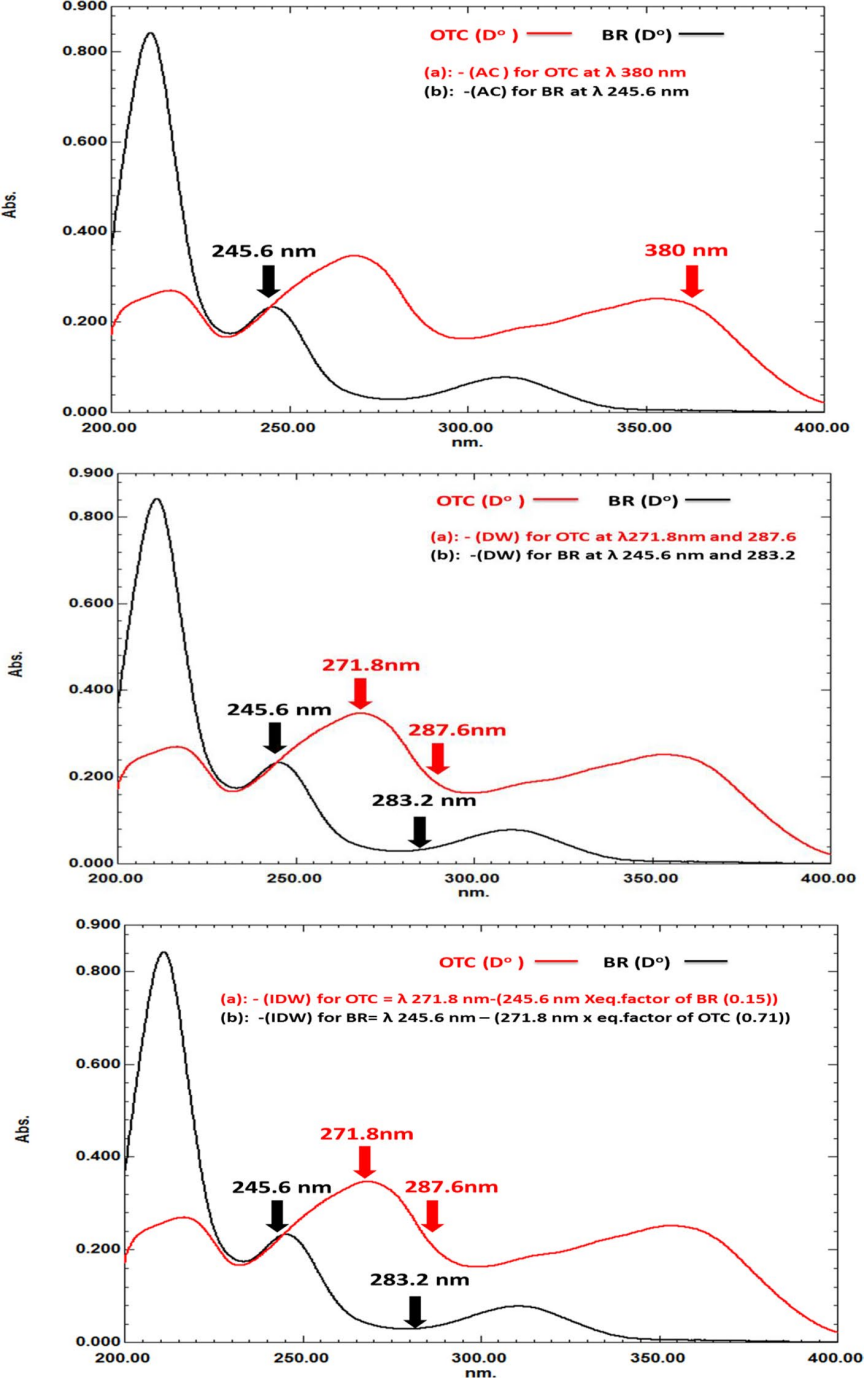
Zero order absorption spectra (10 µg/mL) of each of oxytetracycline and Bromhexine in 0.1 mol/L HCl

Looking at zero order spectra of both OTC and BR, Fig. 2, it was revealed that the spectra of BR and OTC completely overlapped in the range of 200–350 nm, with BR maximum absorbance at 245.6 nm while no contribution for BR at 380 nm.

For OTC, it was determined in the mixture at λ_max_ = 380 nm where BR showed no absorbance at this wavelength and then substitution in its previously computed regression equation.

For BR determination in the mixture, absorption factor of OTC (A_F_ OTC) relating its absorbance at 245.6 and 380 nm was calculated (A _245.6 nm_ /A _380 nm_= 0.97). Then, the absorbance of BR in mixture at 245.6 nm was calculated by multiplying the absorption of the mixture at 380 nm by the calculated absorption factor of OTC to get the absorbance value corresponding to OTC at 245.6 nm. Secondly, subtracting the calculated absorbance value corresponding to OTC from the absorbance of the mixture at 245.6 nm to get BR contribution at 245.6 nm.

$$\eqalign{& {{\rm{A}}_{{\rm{corresponding}}\,{\rm{to}}\,{\rm{OCT}}\,{\rm{at}}\,{\rm{245}}{\rm{.6}}}} = \cr & {{\rm{A}}_{{\rm{F}}\,{\rm{OTC}}}} \times {\rm{A}}\,{\rm{380}}\,{\rm{n}}{{\rm{m}}_{{\rm{(mixture)}}}} \cr}$$

A _of BR at 245.6 nm_ = total mixture absorbance at 245.6 nm – A _corresponding to OTC at 245.6 nm_.

After that, the previously computed equation of BR at 245.6 nm was used to calculate its concentration in the binary mixture.

### Dual wavelength method (DW)

Dual wavelength method provides an effective method for the analysis of a drug in the existence of another interfering drug using their zero order spectra without derivatization [41].

#### Principle

Dual wavelength method depends on pre-selection of two wavelengths [(λ_1_) and (λ_2_)] at which the interfering drug (X) shows the same absorbance (absorption difference between the two wavelengths equal zero (A_Xλ1_ -A_X λ2_ = zero)). In contrary, the drug of interest (Y) shows a significant difference in absorbance. Calibration curve for (Y) is then plotted using the absorbance difference at the selected wavelengths to its corresponding concentrations and then equation is computed. Concentration of (Y) in the binary mixture can be calculated after recording the absorbance difference of the mixture at the selected wavelengths using the calculatd equation. By the same way, component (X) can be determined in the binary mixture without interfering from (Y).

For OTC determination, the absorbance difference between λ_1_ 271.8 nm and λ_2_ 287.6 nm was used.

While for BR, the difference between λ_1_ 245.6 nm and λ_2_ 283.2 nm (as OTC has equal absorbance) was chosen Fig. 2.

### Induced dual wavelength method (IDW)

#### Principle

Unlike dual wavelength method, this technique can be used when the absorbance difference between the two selected wavelengths for the interfering substance is not zero which gives IDW an advantage over DW method [42].

IDW method relies on the prior selection of two wavelengths; (λ_1_) and (λ_2_), at which the interfering drug (X) and the drug of interest (Y) exhibit significant differences in absorbance (absorption difference between the two wavelengths is not equal to zero for any of the measured components).

Firstly, two wavelengths are chosen; λ_1_ ( to determine component Y) and λ_2_ (for component X determination). Both X and Y have considerable absorbance at both λ_1_ and λ_2_.

For analysis of Y:

- Equality factor of pure X is calculated (Fx) which is equal to the ratio of its absorption at λ_1_ to its absorption at λ_2_.
- Calibration curve for pure (Y) is plotted relating the difference in absorption between λ_1_ and λ_2_ (after the multiplication by Fx) to its corresponding concentrations.
- For measuring (Y) in the binary mixture, the absorbance of the mixture at λ_2_ was recorded and then multiplied by the previously calculated (Fx). After that, the obtained value was subtracted from the absorbance of the mixture at λ_1_. This difference was used to calculate concentrations of (y) using the previously computed regression equation.

Component (x) can be determined by the same way using the equality factor of (Y) [F_y_= A_y_ λ_2_/ A_y_ λ_1_] and the regression equation representing the relation between the difference between the absorbance of (X) at λ_2_ and its absorbance at λ_1_ (after multiplication by F_y_) to its corresponding concentrations.

The selected wavelengths for determination of OTC and BR were 271.8 (λ_max_ of OTC) and 245.6 nm (λ_max_ for BR).

For OTC determination in the mixture, equality factor of BR (F_eq_ of BR) was used which is the ratio between absorbance at 271.8 nm to its absorbance at 245.6 nm ((A _271.8 nm_ / A _245.6 nm) =_ 0.15), Then this factor is multiplied by the absorbance of the mixture at 245.6 nm (1). After that, OTC is quantified by subtracting the resulted values from step (1) from the absorbance of mixture at 271.8 nm Fig. 2 as the following equation:

1 $$\eqalign{& {\rm{OTC}}\,{\rm{determination}}\,{\rm{in}}\,{\rm{mixture}}\,{\rm{at}}\,{\rm{271}}{\rm{.8nm}} \cr & {{\rm{A}}_{{\rm{271}}{\rm{.8}}\,{\rm{of}}\,{\rm{mixture}}}} - {\rm{[}}{{\rm{A}}_{{\rm{245}}{\rm{.6}}\,{\rm{nm}}\,{\rm{of}}\,{\rm{mixture}}}}{\rm{X}}\,{{\rm{F}}_{{\rm{eq}}\,{\rm{of BR}}}}{\rm{]}} \cr & \cr}$$

Where F_eq_ of BR = A _BR at 271.8 nm_ / A_BR at 245.6 nm_.

While for BR determination in mixture, equality factor of OTC (F_eq_ of OTC) was computed as the ratio between absorbance at 245.6 nm and 271.8 nm ((A _245. nm_ / A _271.8 nm) =_ 0.71) Fig. 2 as the following equation:

2 $$\eqalign{& {\rm{BR}}\,{\rm{determination}}\,{\rm{in}}\,{\rm{mixture}}\,{\rm{at}}\,{\rm{245}}{\rm{.6}}\,{\rm{nm}} \cr & {{\rm{A}}_{{\rm{245}}{\rm{.6}}\,{\rm{of}}\,{\rm{mixture}}}} - {\rm{[}}{{\rm{A}}_{{\rm{271}}{\rm{.8}}\,{\rm{nm}}\,{\rm{of}}\,{\rm{mixture}}}}{\rm{X}}\,{{\rm{F}}_{{\rm{eq}}\,{\rm{of}}\,{\rm{OTC}}}}{\rm{]}} \cr & \cr}$$

Where F_eq_ of BR = A_OTC_ at 245.6 nm / A_OTC_ at 271.8 nm.

The values obtained from (1) and (2) were used along with the regression equations to calculate the corresponding concentrations of both OTC and BR in the prepared mixtures.

### Spectrum subtraction method (SS)

#### Principle

Spectrum subtraction method is a simple method that relies on using ^0^D absorption spectra. It involves few steps to extract 0D spectra of the proposed drugs [43]. It can be used for the analysis of two partially overlapped drugs (X and Y) at λ_1_ (at which X and Y interferes) and λ_2_ (no contribution from Y).

For drug (X):

- a normalized spectrum is firstly obtained (it is obtained by dividing each spectrum by its corresponding concentration). After that, the mixture of (X and Y) is divided by the normalized spectrum of (X) to get a plateau region.
- absorbance at λ_1_ in the plateue is recorded, then multiplied by the normalized spectrum of (X) to get the parent spectrum of (X) in mixture.

For drug (Y):

a-it can be determined by subtracting the resolved spectrum of (X) from the total spectrum of the mixture, and then the absorbance of the resulted spectrum of (Y) is recorded at λ_2_.

**Fig. 3:**
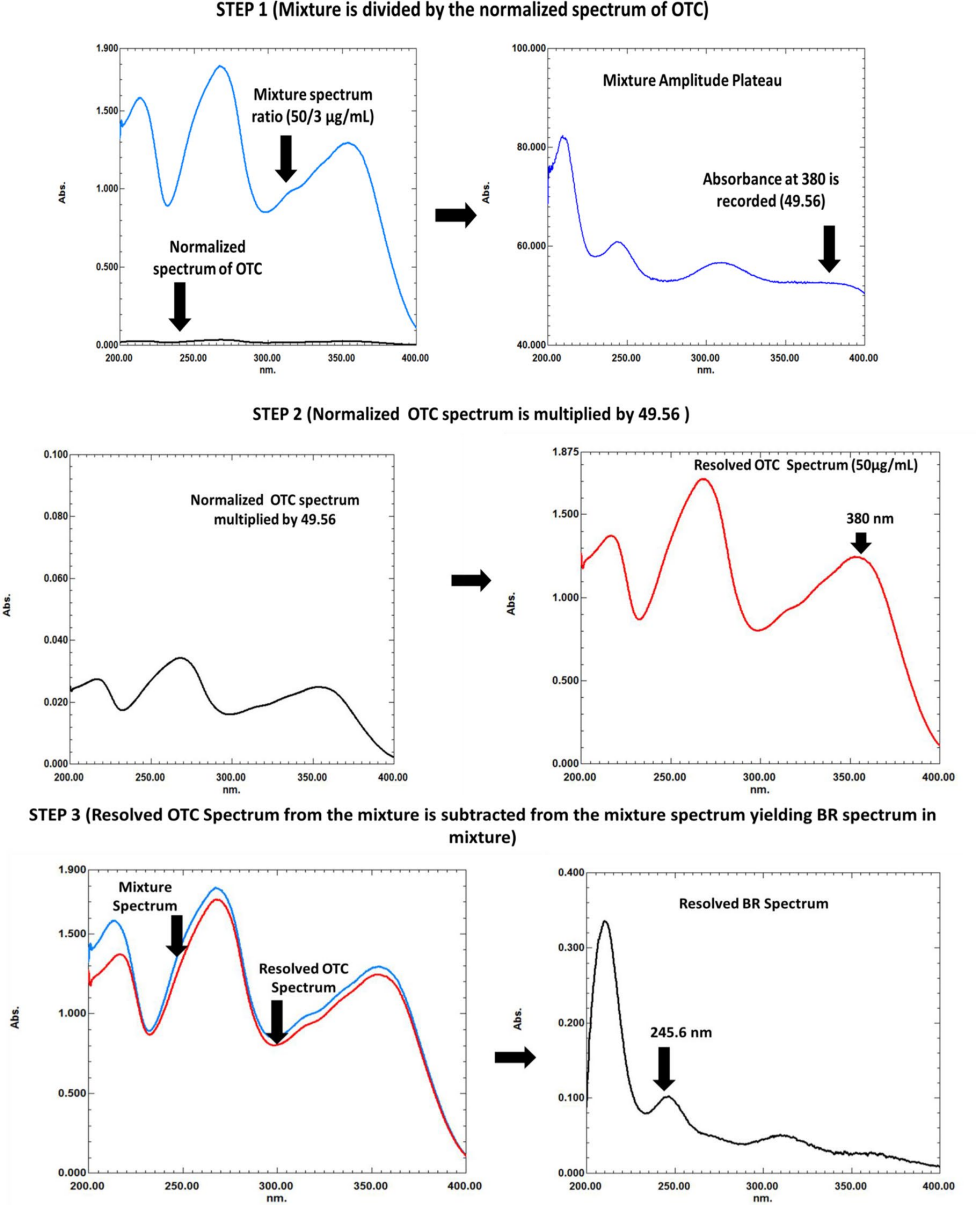
Spectrum subtraction method for the determination of both OTC and BR in D^0^ spectra

For OTC determination, the spectrum of the mixture was divided by the normalized spectrum of OTC to obtain a plateau region. The absorbance at 380 nm in the plateau was then recorded and multiplied by the normalized spectrum of OTC to extract ^0^D spectrum of OTC in the mixture from which it can be directly determined using the absorbance value at 271.8 nm (λ _max_). Figure 3 For BR determination, the extracted ^0^D of OTC was subtracted from the total mixture spectrum resulting in ^0^D spectrum of BR that can be measured at 245.6 (λ _max_), Fig. 3.

### Method validation

The proposed methods have been validated in accordance with ICH standards [44]. The following parameters were determined: linearity and range, accuracy, precision (repeatability and intermediate precision), specificity and limit of detection and limit of quantitation.

#### Linearity

Upon applying the proposed methods linear relationship were obtained over the range of 2–50 µg/mL and 1–30 µg/ mL for OTC and BR, respectively. All regression parameters were computed and demonstrated in Table 1. All the gotten values were > 0.999 ensuring linearity of the methods over the tested ranges.

**Table 1:** Regression and validation parameters of the proposed methods for determination of oxytetracycline and bromhexine

Methods	OTC							BR							
Parameters	AC		SS	DW		IDW		AC	SS		DW			IDW	
Range (µg/mL)	2–50							1–30							
Slope	0.0240	0.0240			0.0140	0.0284		0.023		0.023		0.0203			0.0217
Intercept	0.0017	0.0017			0.0005	− 0.0001		0.005		0.005		-0.0018			− 0.0208
Correlation coefficient (r)	0.9998	0.9998			0.9996	0.9998		0.9999		0.9999		0.9999			0.9999
Accuracy ^(a)^ Recovery Mean ±%RSD	99.53 ± 0.81	99.5 3± 0.81			100.43± 0.67	99.42 ± 0.94		98.95± 1.01		98.95 ± 1.01		100.35 ± 1.20			98.88 ± 0.96
Precision (RSD %) Repeatability ^(b)^ Intermediate precision ^(c)^	0.91 1.71	0.91 1.71			0.80 1.11		1.37 1.44	1.25 1.36		1.25 1.36			1.54 1.73		1.22 1.26
LOD ^(d)^	0.618		0.618		0.560	0.554		0.186		0.186		0.219			0.173
LOQ ^(d)^	1.854		1.854		1.707	1.687		0.563		0.563		0.663			0.525

(a) Average recovery of 3 concentration ratios measured in triplicates

(b) Repeatability ; 3 concentrations for each drug ; (15, 35, 45 µg/mL) for OTC and (3, 20, 25 µg/mL) for BR, each was determined three time on the same day

(c) Intermediate precision ; 3 concentrations for each drug ; (15, 35, 45 µg/mL) for OTC and (3, 20, 25 µg/mL) for BR, each determined on three different days

(d) LOD = 3.3*SD/Slope LOQ = 10*SD/Slope, as the SD of the intercept and slopes of the calibration curves

#### Accuracy

The accuracy of the developed methods was examined using three different samples (10, 20, 40 µg/mL) for OTC and (10, 15, 20 µg/mL) for BR and each single sample was analyzed three times. The concentrations were calculated from the related regression equations and results were expressed by mean recovery and relative standard deviation Table 1. All values were close to 100 where the accuracy of OTC ranged from 99.42 ± 0.67–100.34 ± 0.94 and for BR ranged from 98.88 ± 0.96 -100.35 ± 1.20. Further studies for the accuracy of the proposed methods were established by application of standard addition approach, where known pure samples of OTC and BR were added to the pharmaceutical dosage form Table 2. It was tested on three concentration levels. All the obtained values revealed the validity and the accuracy of the method, additionally; it ensured the absence of excipient interference.

**Table 2:** Determination of oxytetracycline and bromhexine in Oxyclear^®^ injection solution by the proposed spectrophotometric methods and the application of the standard addition technique

Taken µg/mL				OTC				BR			
				% Found				% Found			
				AC	SS	DW	IDW	AC	SS	DW	IDW
Dosage form Oxyclear^®^ (batch number 190317)	OTC	BR									
	25.00	1.50		101.55	100.81	98.14	101.89	101.29	101.17	101.90	97.66
				100.21	100.64	102.14	101.37	98.12	98.43	98.77	99.16
				99.55	100.14	100.14	101.77	99.85	99.64	100.13	98.33
				99.05	99.23	97.02	99.75	102.16	101.54	98.25	100.05
				97.55	98.78	98.01	97.87	99.27	99.52	101.80	101.66
				102.21	101.78	98.85	100.44	100.72	100.84	99.12	101.33
	**(Mean)**			**100.02**	**100.23**	**99.05**	**100.51**	**100.23**	**100.19**	**99.99**	**99.69**
	**RSD%**			**1.68**	**1.09**	**1.85**	**1.52**	**1.45**	**1.19**	**1.56**	**1.62**
Standard addition on dosage form ratio 25/1.5 µg/mL	Pure ratio added µg/mL			OTC				BR			
				% Found				% Found			
	OTC		BR	AC	SS	DW	IDW	AC-SS	SS	DW	IDW
	15.00		3.00	99.52	99.23	101.90	101.27	101.60	101.42	101.30	99.75
	20.00		5.00	99.64	99.79	100.00	101.71	101.65	100.91	100.00	99.16
	25.00		10.00	101.38	100.88	100.57	101.57	99.74	100.33	100.89	99.5
	**(Mean)**			**100.18**	**99.97**	**100.82**	**101.51**	**100.99**	**100.89**	**100.73**	**99.47**
	**RSD%**			**1.04**	**0.84**	**0.96**	**0.22**	**1.08**	**0.55**	**0.66**	**0.29**

#### Precision

Three different concentrations of OTC and BR (15, 35, 45 µg/mL) and (3, 20, 25 µg/mL), respectively were analyzed in triplicates in the same day and on three constitutive days for assuring repeatability and intermediate precision, in order. The obtained results were expressed in relative standard deviation (% RSD) and they are given in Table 1. The entire obtained % RSD were < 2% confirming good precision of the proposed methods.

#### Specificity

Specificity of the developed methods was tested by their application for analysis of six prepared laboratory prepared mixtures that contain different ratios of OTC and BR within their linearity ranges. Results were expressed as mean recoveries and % RSD. The obtained recoveries were within the acceptable values confirming no interference between the two drugs Table 3.

**Table 3:** Determination of oxytetracycline and bromhexine in the laboratory prepared mixtures by the proposed spectrophotometric methods

Taken µg/mL		OTC				BR			
		% Found				% Found			
		AC	SS	DW	IDW	AC	SS	DW	IDW
OTC	BR								
*25	1.5	100.60	99.91	100.60	99.41	100.51	100.13	98.42	98.00
20	20	101.30	100.24	100.60	101.00	99.19	99.74	100.37	98.57
40	2	101.50	98.67	100.80	101.40	98.44	99.22	99.21	98.25
*50	3	101.20	97.39	100.10	100.60	97.26	98.31	100.60	97.50
50	5	100.40	100.97	99.38	101.00	101.60	100.76	98.40	98.57
30	10	100.00	99.74	99.20	100.00	99.20	99.45	100.00	99.00
**(Mean)**		**100.83**	**99.49**	**100.11**	**100.56**	**99.36**	**99.6**	**99.50**	**98.31**
**%RSD**		**0.92**	**0.92**	**0.71**	**0.99**	**1.21**	**1.21**	**1.25**	**1.19**

*The ratio of the two drugs in their combined dosage form

Moreover, the proposed methods were applied to the dosage form sample (25/1.5 µg/mL). The obtained results ranged from 99.05 to 100.51% for OTC and ranged from 99.69 to 100.23% for BR assuring no interference from the additives. Table 2.

#### Limit of detection and quantitation

High sensitivity of the developed approaches was confirmed by low values of both LOD and LOQ for both OTC and BR, respectively as presented in Table 1. SD of the intercepts and slopes of the calibration curves were used to calculate both LOD and LOQ using the following equations :

LOD = 3.3*SD/Slope.

LOQ = 10*SD/Slope.

All the obtained results ensured sensitivity of the suggested methods.

### Statistical analysis

A statistical comparison was carried out between the results produced by the developed methods and the results of the reported HPLC method [31] as shown in Table 4. It also was found that the calculated t-values and F-values were less than that of the reported method, showing that there is no significant difference between the proposed and reported method.

**Table 4:** Statistical comparison of the results obtained by applying the proposed spectrophotometric methods and the reported method for determination of oxytetracycline and bromhexine

items	OTC			BR			Reported HPLC method [31]	
	AC-SS	DW	IDW	AC-SS	DW	IDW	OTC	BR
Mean	99.53	100.34	99.42	98.95	100.35	98.88	99.63	100.12
SD	1.22	1.10	1.21	1.50	1.14	1.12	0.96	1.08
N	9	9	9	9	9	9	9	9
Student’s t-test (2.131)	1.75	1.75	1.76	1.76	1.75	1.73	-	-
F-test (3.438)	1.27	1.14	1.26	1.38	1.05	1.03	-	-

### Comparative study between the proposed spectrophotometric methods and the reported HPLC method [31] regarding their greenness profile

Five metric systems namely National Environmental Methods Index (NEMI) [34], Modified NEMI [35], Analytical Eco-scale [36], Green Analytical Procedure Index (GAPI) [37] and Analytical GREEnness (AGREE) [38] for evaluating the developed spectrophotometric methods and the reported HPLC methods. Results of green profile are presented in Table 5.

**Table 5:**
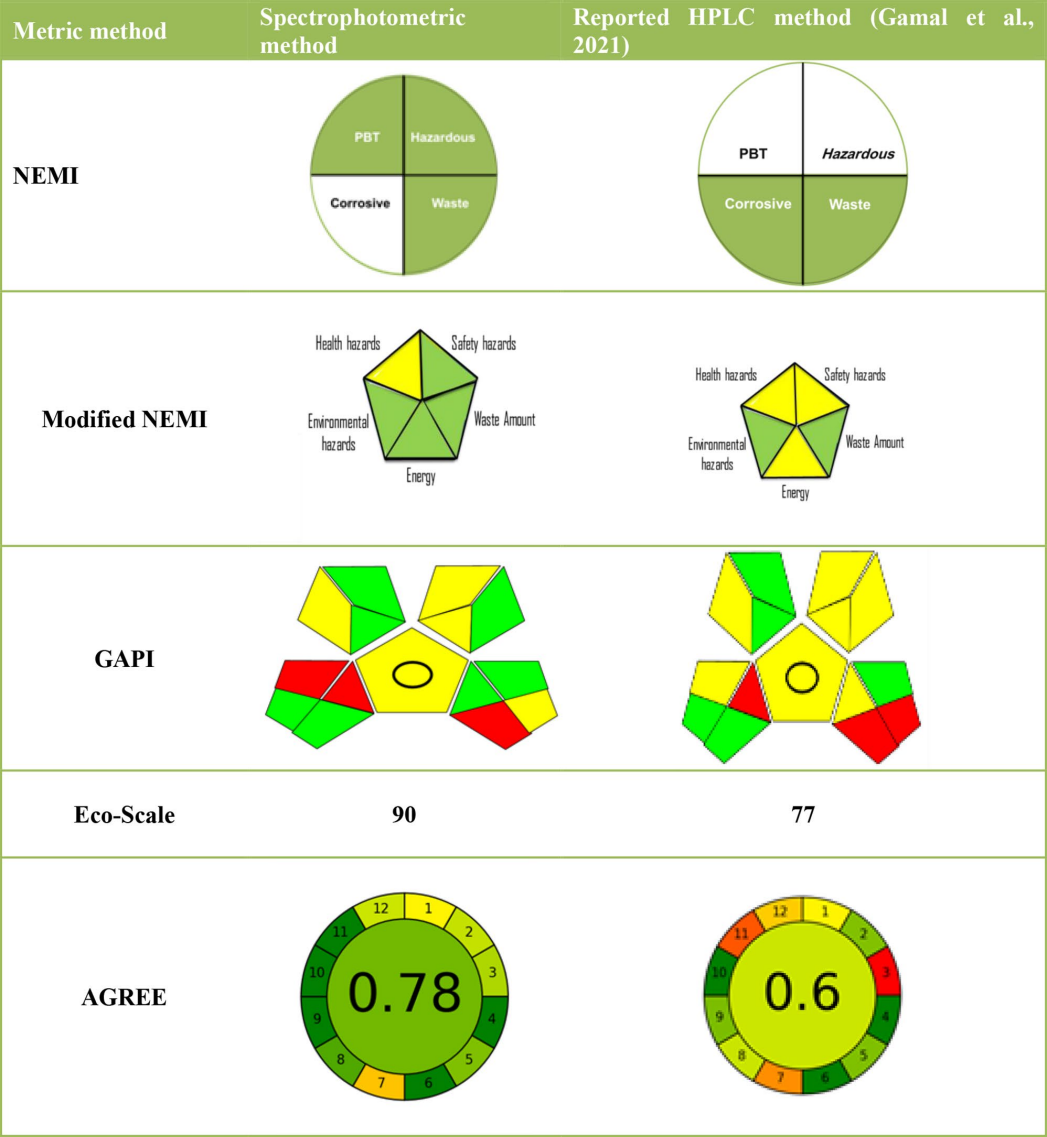
Green profile for the developed spectrophotometric methods and the reported TLC and HPLC methods

National Environmental Methods Index (NEMI), it is a metric system used as a qualitative evaluator which deals only with chemicals and reagents. It consists of four sectioned pictogram divided as the following; PBT (persistent, bio accumulative and toxic), Hazardous, Corrosive and waste. Spectrophotometric methods showed three green sections, this is due to the usage of hydrochloric acid which is not listed in EPA’s TRI list and RCRA list so it is not considered as a PBT or hazardous solvent keeping the upper two sections green .On the other hand, pH of 0.1 mol/L HCl which is reported to be corrosive. Additionally, the waste for one sample was 10 mL so the waste generated was found to be less than 50gm keeping the waste section colored green. For the reported HPLC method [31], it showed one green sections only due to usage of acetonitrile and methanol (PBT) and triflurocacetic acid (Hazardous and corrssive), Table 5.

Regarding to the modified NEMI tool, it was found to take an advantage over the NEMI system by taking into consideration the energy of the instruments. It is a five sectioned pictogram represents health hazards (NFPA scores for health hazards), safety hazards (NFPA scores for flammability hazards), environmental hazards, energy, and waste amount. Each section consists of three sections colored; green, yellow or red which indicates the greenness of each section. Spectrophotometric methods had four green fields (Environmental hazards, safety hazards, energy and waste amount) with one yellow field (Health hazards). On the other hand, the reported HPLC methods [31] showed three yellow fields (Health hazards, Safety hazards, Energy) and two green fields (Environmental hazards and waste amount) giving the developed spectrophotometric methods the advantage over the reported ones.

For GAPI metric system, it is a semi quantitative tool that deals with different parameters including sample preparation, reagents used and instrumentation. Spectrophotometric methods resulted in seven green, five yellow and three red sections (due to moderately high solvent consumption for preparation, waste generated and health hazards of HCl), For the HPLC reported method [31], it showed six green and four yellow and three red areas. The developed spectrophotometric methods showed higher green areas due to the lower energy consumption in spectrophotometer instrument and less sample preparation steps giving them the advantage of being greener than reported method.

For Eco-scale system, it is also a semi quantitative tool that took into consideration the hazard effects of each solvent singly. For spectrophotometric methods, the use of low number of solvents solvents (hydrochloric acid only) and lower energy consumption gave lower subtracted penalty points (10 PP) than the reported ones which scored 23 PP [31]. This is due to the higher energy needed and higher hazards solvents used in the reported method compared to spectrophotometric method ensuring that the developed spectrophotometric methods can be used as greener alternatives for the reported method.

Concerning to AGREE metric, it is a quantitative tool depends on converting the 12 principles of green analytical chemistry into a score ranged from 0 to 1. For the developed spectrophotometric methods, the overall score for AGREE system was 0.78 while for HPLC method [31] it scored 0.6, this is due to the production of higher waste amounts, higher consumption of toxic solvents and higher energy needed for these reported methods.

It is obvious that every metric method helped to reveal a green side by providing certain information about specific issue concerning the developed and the reported methods giving a complete vision on the comparative study between them. Hence, it is advisable to use more than one metric system to evaluate any method of analysis. The used tools confirmed the superiority of the developed methods over the reported one. Details are given in Table S.2–S.6.

## Conclusion

Successfully the developed spectrophotometric methods proved the ability for determination of OTC and BR in their binary mixture in the pure form and in the pharmaceutical dosage form without any previously separation steps. The suggested methods are fast, accurate, precise, selective, and reproducible. Also the developed methods were able to resolve the overlapped drugs with few steps in zero order spectra without any further dervitization steps. Validation of the developed methods was applied according to the ICH guidelines giving acceptable results. Furthermore, the application of green chemistry principles has evaluated the environmental impact of the developed methods using five different metrics: NEMI, Modified NEMI, Analytical Eco-scale, GAPI, and AGREE which demonstrated that they are not only efficient but also environmentally responsible. The developed methods proved that they can be used effectively for the routine analysis of the investigated drugs in QC laboratories.

## Electronic supplementary material

Below is the link to the electronic supplementary material.

Supplementary Material 1

## Data Availability

Data is provided within the manuscript or supplementary information files.
