# Supplementary material for: Analysis of binary mixture of oxytetracycline and bromohexine in their combined veterinary formulation by four simple spectrophotometric methods with greenness assessment
Source: BMC Chem. 2024 Oct 3;18(1):192. doi: 10.1186/s13065-024-01296-y (PMC11451190; doi:10.1186/s13065-024-01296-y)
Supplement: Supplementary file 1 — Supplementary Material 1 [file 13065_2024_1296_MOESM1_ESM.docx]

**Table S.1**: Laboratory prepared mixtures (preparation in 10 mL)

| **OTC** | | **BR** | |
| --- | --- | --- | --- |
| **Ratio** | **Taken Volume in mL (100 µg/mL)** | **Ratio** | **Taken Volume (100 µg/mL)** |
| ***25** | 2.5mL | ***1.5** | 0.15mL |
| **20** | 2 mL | **20** | 2 mL |
| **40** | 4 mL | **2** | 0.2mL |
| ***50** | 5 mL | ***3** | 0.3 mL |
| **50** | 5 mL | **5** | 0.5mL |
| **30** | 3 mL | **10** | 1 mL |

**Table S.2**: NEMI metric system for the assessment of Spectrophotometric methods and the TLC and HPLC reported methods.

| - | PBT reagents | Non PBT reagents | Hazardous reagents (RCRA included solvents) | Nonhazardous (RCRA non included solvents) | corrosive | waste | Pictogram |
| --- | --- | --- | --- | --- | --- | --- | --- |
| Spectrophotometeric methods | Hydrochloric acid | - | Hydrochloric acid | - | pH=1  (0.1N HCl) | Generated waste =10 ml < 50mL | 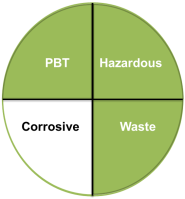 |
| Reported HPLC | Methanol | Trifluroacetic acid- acetonitrile | Trifluroacetic acid  acetonitrile | - | 2<PH < 12 | Generated waste =22ml < 50ml | 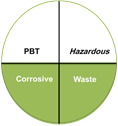 |

**Table S.3:** Modified NEMI metric system for the assessment of Spectrophotometric methods and the TLC and HPLC reported methods

|  | **Reported HPLC** | | | **Spectrophotmeteric methods** | | |
| --- | --- | --- | --- | --- | --- | --- |
| **Category** | **Green** | **Yellow** | **Red** | **Green** | **Yellow** | **Red** |
| **Health Hazard**  **According to NFPA health hazards score :**  **-Green :0-1**  **-Yellow:2-3**  **-Red: 4-5** |  | **trifluroacetic acid=1**  **Acetonitrile=2**  **Methanol=2** |  |  | **Hydrochloric acid=3** |  |
| **Safety hazard**  **According to NFPA flammability hazards score :**  **-Green :0-1**  **-Yellow:2-3**  **-Red: 4-5** |  | **trifluroacetic acid=0**  **Acetonitrile= 3**  **Methanol= 3** |  | **HCl= 0** |  |  |
| **Environmental hazard**  **-Green: If the Environmental hazard is less than 50 g.**  **-Yellow:** **If the Environmental hazard is ranged from 50-250g**  **-Red: If the Environmental hazard is higher than 250g.** | **Environmental hazard is less than 50** |  |  | **Environmental hazard is less than 50** |  |  |
| **Energy** |  | **Instrumental method HPLC <1.5** |  | **Instrumental method spectrophotometer <0.01** |  |  |
| **Waste amount**  **The total waste was less than 50 g or mL for one sample analysis.** | **Less than 50 g** |  |  | **Less than 50 g** |  |  |
| ***Pictograms*** | **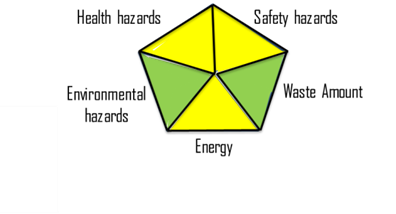** | | | **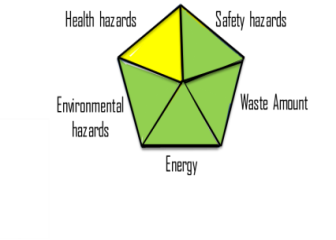** | | |

**Table S.4:** GAPI metric system for the assessment of Spectrophotometric methods and the TLC and HPLC reported methods.

| **Sample Preparation** | | | | | | | | | | | |
| --- | --- | --- | --- | --- | --- | --- | --- | --- | --- | --- | --- |
|  | Collection | preservation | transport | | storage | Type of method | | Scale of extraction | | Solvents/reagents used | Additional treatments |
| **Spectrophotometric method** | Off-line | None | None | | Under normal conditions | Simple procedures | | Micro-extraction | | green solvents/ reagents | None |
| **Reported method** | Off-line | None | None | | Under normal conditions | Simple procedures | | Micro-extraction | | Non-green solvents/ reagents | None |
| **Reagents and Solvents** | | | | | | | | | | | |
|  | Amount | | | Health Hazards | | | | | Safety Hazards | | |
| **Spectrophotometric method** | 10-100 mL (10-100 g) | | | Moderately toxic; could cause temporary incapacitation; NFPA = 2 or 3. | | | | | Highest NFPA flammability or instability score of 0 or 1. No special hazards. | | |
| **Reported method [19]** | 10-100 mL (10-100 g) | | | Moderately toxic; could cause temporary incapacitation; NFPA = 2 or 3. | | | | | Highest NFPA flammability or instability score = 2 or 3, or a special hazard is used. | | |
| **Instrumentation** | | | | | | | | | | | |
|  | Energy | | | Occupational Hazards | | | Waste | | Waste Treatment | | |
| **Spectrophotometric method** | <= 0.1 kWh per sample | | | Hermetic sealing of the analytical process | | | 1-10 mL (1-10 g) | | No treatment | | |
| **Reported method [19]** | <= 1.5 kWh per sample | | | Hermetic sealing of the analytical process | | | > 10 mL (>10 g) | | No treatment | | |
| **Pictograms** | | | | | | | | | | | |
| **Spectrophotometric method** | 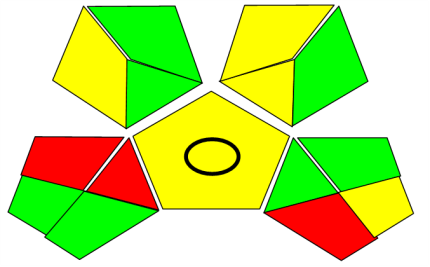 | | | | | | | | | | |
| **Reported method [19]** | 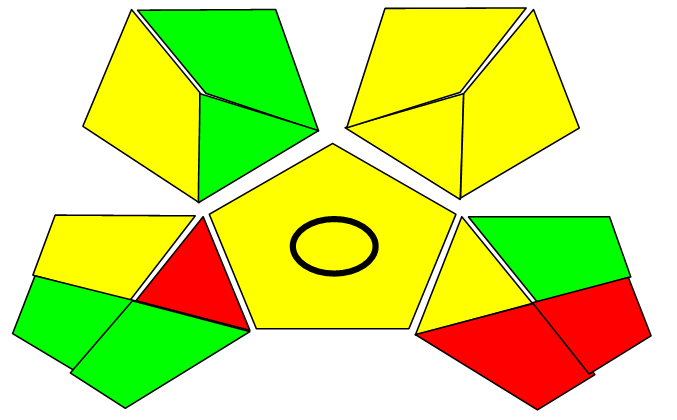 | | | | | | | | | | |

**Table S.5:** AGREE metric system for the assessment of Spectrophotometric methods and the TLC and HPLC reported methods.

| Parameters | Spectrophotometric Method | HPLC method |
| --- | --- | --- |
| Principle 1. Direct Analytical Techniques Should Be Applied to Avoid Sample Treatment. (Sampling Procedure) | Off-line Procedure | Off-line Procedure |
| Principle 2. Minimal Sample Size and Minimal Number  of Samples Are Goals. (Amount of sample in either gm or ml) | 1.25 mL | 1 mL |
| Principle 3. In Situ Measurements Should Be  Performed. (Positioning of analytical device) | o-line Procedure | on-line Procedure |
| Principle 4. Integration of Analytical Processes and Operations Saves Energy and Reduces the Use of Reagents. (sample steps preparation) | Fewer than 3 | Fewer than 3 |
| Principle 5. Automated and Miniaturized Methods  Should Be Selected. (Degree of automation and sample preparation) | Semi-automated-none or miniaturized | Semi-automated-none or miniaturized |
| Principle 6. Derivatization Should Be Avoided. (Derivatization agents) | None | None |
| Principle 7. Generation of a Large Volume of Analytical Waste Should Be Avoided and Proper Management of Analytical Waste Should Be Provided. (amount of waste in gm or ml) | 10 mL | 22 mL |
| Principle 8. Multianalyte or Multiparameter Methods Are Preferred versus Methods Using One Analyte at a Time. (number of analytes analysed in single run / samples analysed per hour) | 2/20 | 3/5 |
| Principle 9. The Use of Energy Should Be Minimized.(most energy-intensive technique used in method) | UV-Vis spectrometry | LC |
| Principle 10. Reagents Obtained from Renewable  Source Should Be Preferred. (Types of reagents) | Some reagents are bio-based | all reagents are bio-based |
| Principle 11. Toxic Reagents Should Be Eliminated or  Replaced.( Involvement of toxic reagents - number of toxic reagents) | No | Yes – 12.8  (methanol and acetonitrile) |
| Principle 12. The Safety of the Operator Should Be  Increased. (Threats which are not avoided) | Toxic to aquatic life-Corrosive  (Hydrochloric acid) | Toxic to aquatic life (trifluroacetic acid)  Highly flammable(methanol-acetonitrile)  Corrosive (trifluroacetic acid) |
| Clock like AGREE graph | 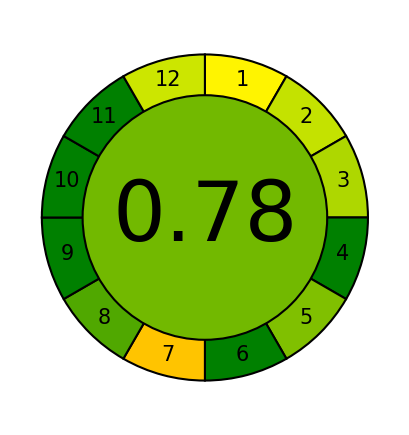 | 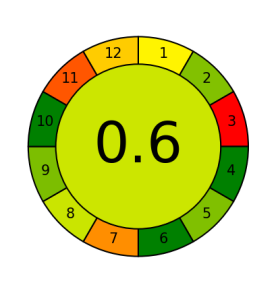 |

| Reported HPLC | | | | | | | |
| --- | --- | --- | --- | --- | --- | --- | --- |
| Parameters | Reagents | | | Instrument | | | |
|  | methanol | Trifluroacetic acid 0.1% | Acetonitrile | Energy | Occupational hazard | waste | |
|  |  |  |  |  |  | Waste generated | Waste treatment |
|  |  |  |  | ≤1.5 kWh per sample | Analytical process hermetization | >10 mL | No waste treatment |
| Consumed volume = run time x flow rate x solvent percentage in mobile phase | 10 | 0.01 | 2.8 |  |  |  |  |
| Subtotal PP (solvent < 10ml) | 1 | 1 | 1 |  |  |  |  |
| Signal word (Danger = 2PP , Warning = 1PP) | 2 | 2 | 2 |  |  |  |  |
| No. of pictogram | 2 | 3 | 2 |  |  |  |  |
| PP of solvent  = subtotal PP x signal words PP x no.pictograms | 4 | 6 | 4 | 1PP | 0 PP | 5 | 3 |
| Penalty points summation | **14** | | | **9** | | | |
| Total Penalty points | 23 | | | | | | |
| **Analytical Eco-scale score = 100-23 = 77** | | | | | | | |

| Spectrophotometric method | | | | | |
| --- | --- | --- | --- | --- | --- |
| Parameters | Reagents | Instrument | | | |
|  | Hydrochloric acid | Energy | Occupational hazard | waste | |
|  |  |  |  | Waste generated | Waste treatment |
|  |  | ≤0.1 kWh per sample | Analytical process hermetization | >10 mL | No waste treatment |
| Consumed volume | 0.083ml of conc HCl |  |  |  |  |
| Subtotal PP (solvent < 10ml) | 1 |  |  |  |  |
| Signal word (Danger = 2PP , Warning = 1PP) | 2 |  |  |  |  |
| No. of pictogram | 2 |  |  |  |  |
| PP of solvent  = subtotal PP x signal words PP x no.pictograms | 4 | 0PP | 0 PP | 3 PP | 3PP |
| Penalty points summation | **4** | **6** | | | |
| Total Penalty points | 10 | | | | |
| **Analytical Eco-scale score = 100-10 = 90** | | | | | |

**Table S.6:** Eco-Scale metric system for the assessment of Spectrophotometric methods and the HPLC reported methods.
